# A Review of Frameworks for Developing Environmental Health Indicators for Climate Change and Health

**DOI:** 10.3390/ijerph8072854

**Published:** 2011-07-13

**Authors:** Tammy Hambling, Philip Weinstein, David Slaney

**Affiliations:** 1 Institute of Environmental Science & Research Limited, 34 Kenepuru Drive, Porirua 5022, New Zealand; E-Mail: david.slaney@esr.cri.nz; 2 School of Population Health, University of Queensland, Herston Road, Herston, Queensland 4600, Australia; 3 Barbara Hardy Institute, University of South Australia, Adelaide, South Australia 5000, Australia; E-Mail: philip.weinstein@unisa.edu.au

**Keywords:** climate change, DPSEEA, environmental health, frameworks, indicators, monitoring, policy

## Abstract

The role climate change may play in altering human health, particularly in the emergence and spread of diseases, is an evolving area of research. It is important to understand this relationship because it will compound the already significant burden of diseases on national economies and public health. Authorities need to be able to assess, anticipate, and monitor human health vulnerability to climate change, in order to plan for, or implement action to avoid these eventualities. Environmental health indicators (EHIs) provide a tool to assess, monitor, and quantify human health vulnerability, to aid in the design and targeting of interventions, and measure the effectiveness of climate change adaptation and mitigation activities. Our aim was to identify the most suitable framework for developing EHIs to measure and monitor the impacts of climate change on human health and inform the development of interventions. Using published literature we reviewed the attributes of 11 frameworks. We identified the Driving force-Pressure-State-Exposure-Effect-Action (DPSEEA) framework as the most suitable one for developing EHIs for climate change and health. We propose the use of EHIs as a valuable tool to assess, quantify, and monitor human health vulnerability, design and target interventions, and measure the effectiveness of climate change adaptation and mitigation activities. In this paper, we lay the groundwork for the future development of EHIs as a multidisciplinary approach to link existing environmental and epidemiological data and networks. Analysis of such data will contribute to an enhanced understanding of the relationship between climate change and human health.

## 1. Introduction

Over recent years there has been a growing body of evidence and consensus that global climate change is occurring [1]. The Fourth Assessment by the Intergovernmental Panel on Climate Change (IPCC) states that the likely range for global average air warming [calculated from the six emissions scenarios described in the Special Report on Emissions Scenarios (SRES)] will be between 1.1 °C to 6.4 °C by the end of the 21^st^ century. The SRES future emission scenarios range in character from a more ecologically friendly situation to scenarios of rapid economic growth with an emphasis on fossil fuels [2]. There has also been a significant amount of peer-reviewed science published since the IPCC’s Fourth Assessment in 2007. These recent revisions of projected changes now suggest that the IPCC projections may be rather conservative [3,4]. That said, for the purposes of our paper the term climate change includes both human induced and natural changes, as well as climate variability and extreme weather events.

Although the impacts and consequences of climate change are likely to be many and complex, climate change is already affecting human health and well-being. Health effects may be direct and include mortality and morbidity from extreme heat, cold, droughts, or storms, and from changes in air and water quality caused by changes in temperature, rainfall, and other climate variables. Alternatively, health effects may be indirect, for example through changes in the ecology of infectious diseases or impacts on air quality and landscapes that can affect human well-being [5–7].

Two World Health Organization (WHO) reports on climate change and human health highlight that one of the aims of climate change and health monitoring should be to detect early health impacts of climate change [7,8]. While much health-impacts research focuses on future risk, it is also important to undertake empirical studies referring to the recent past and present. Disease incidence data are needed to provide a baseline for epidemiological studies [8]. The four main criteria for selecting priority diseases for surveillance are climate sensitivity, knowledge of transmission cycle, significance as a threat to public health, and logistic constraints [7]. More recently there has been a call to increase the focus on applied research to lessen the health risks associated with climate, through improved risk assessment and by designing effective actions [9]. There has also been an explicit request in the WHO workplan [10] where WHO’s Member States have asked the WHO to implement on climate change and health—Action 3.6: Identify and develop indicators to monitor climate change-related health outcomes within surveillance systems.

In the 2001 WHO report, the need for a two-pronged approach was identified to detect and respond to the early impacts of climate change on health: (i) through informed monitoring of appropriate indicators at a national level; and (ii) through specifically targeted research projects that will strengthen the case for mitigation and adaptation to climate change, and test the plausibility of any research methods and tools developed [7].

Due to the multidisciplinary nature of research, the complexity of many interacting factors, and the numerous uncertainties involved in studying the impacts of climate change on health, there are significant research gaps in this field at present. Assessment of health outcomes related to climate change is complex as it must accommodate multiple uncertainties. The contribution of factors such as socioeconomic development, land-use, urbanization, or globalization to the transmission of diseases can be more important than climate change alone, therefore making quantification complex [6,11]. In addition to the multiple uncertainties already discussed are other critical factors, such as the human dimensions of global change, including institutional sustainability and adaptive capacity to respond to such changes. However, the WHO in the Global Health Risks report estimates that in 2004, 0.2% of all deaths globally were attributable to global climate change [12]. Based on a recent review of published work by Semenza and Menne [6] the European Centre for Disease Prevention and Control identified the need to develop a blueprint for an environmental and epidemiological network to link existing resources. Integration and analysis of such data would benefit and advance the understanding of the relationship between diseases and climate change, and environmental health indicators (EHIs) are one tool that can achieve this (e.g., Kjellström and Corvalán, WHO [13,14]).

Given the complexity of environmental health issues, it is important to use a systematic structured framework to select and develop a relevant range of EHIs and to enable consistent monitoring and interpretation. Various frameworks have been designed for developing indicators and assessing vulnerability, and the framework’s suitability will vary depending on the issue of concern. In this review we evaluated frameworks that had environmental and health components, or were indicator-based and had environmental components, but we did not evaluate the utility of indicator-based tools beyond EHIs. This paper lays the groundwork for the future development of EHIs as a multidisciplinary approach to link existing environmental and epidemiological data and networks. Analysis of such data will contribute to an enhanced understanding of the relationship between climate change and human health.

## 2. Climate Change and Environmental Health Indicators

New threats to human health, particularly the emergence and spread of diseases, are becoming major issues associated with components of global environmental change [15–17]. Contributing to these human health threats are the roles that climate variability and change and extreme weather events play in altering disease risk [5,18–20]. The roles of climate variability and change are important as these processes will compound the already significant burden of diseases (e.g., vector-, food-, and water-borne diseases and respiratory diseases) on national economies and public health. Authorities need to be able to assess, anticipate, and monitor human health vulnerability to climate variability and change to plan for, or implement action to avoid, these eventualities. Environmental health indicators provide a tool to assess and monitor human health vulnerability, to aid in the design and targeting of interventions, and measure the effectiveness of climate change adaptation activities.

At present no fully functioning EHI programs focus on climate change, however, several programs are in development. The WHO Europe, through a project called “Climate Change, Environment and Health Action Plan and Information System”, is developing EHIs and expanding the scope of their Environment and Health Information System to enable monitoring and assessment of environmental health issues related to climate change [21]. The United States Centers for Disease Control and Prevention (CDC) National Environmental Public Health Tracking Network is developing climate change indicators [22]. Another project by English *et al.* [23] has completed a review of EHIs of climate change for the United States.

Climate-specific EHIs aim to enable the identification and analysis of the general consequences of climate variation and change on human health, and enable the inclusion of the myriad of possible impacts on natural and constructed systems that could have secondary or incidental health effects. They will also provide baseline information for assessing and monitoring temporal and spatial variability of risks, enabling projection scenarios of how the current situation may evolve.

## 3. Environmental Health Indicators

An EHI is defined as:

*“An expression of the link between environment and health targeted at an issue of specific policy or management concern and presented in a form, which facilitates interpretation for effective decision making”* [24].

A primary characteristic of EHIs is that they provide information about a scientifically based linkage between the environment and health [13]. They enable the conversion of data to information by summarizing the complex relationships between the environment and health, and presenting them in a form that is more easily interpreted by the end-users, for example, policy makers [25].

The development of good EHIs is challenging because they must satisfy multiple criteria in order to be effective. Several sets of criteria have been developed for EHIs with the following genera l criteria commonly agreed on—they should be scientifically valid or credible, have clear relevance and utility, and be practical [25]. More specific criteria include that EHIs must provide a relevant and meaningful summary of the conditions of interest in a way that meets the requirements of the end-users (who are often not experts in the subject), and be transparent, testable, scientifically sound, robust and sensitive to real changes in the conditions they measure. They should also utilize routinely collected data and, importantly, they must be cost-effective to apply and produce [14].

Climate change environmental health indicators (CCEHIs) are more defined EHIs that are specifically aimed at monitoring the effects of climate change on health. They can be defined as “an expression of the link between climate change and health, targeted to a specific policy or management concern and presented in a form, which facilitates interpretation for effective decision-making” (adapted from Corvalán *et al.* [7]).

A CCEHI is based on a known relationship between climate, an environmental exposure, and health. They have an important temporal component as they are used to detect change over time, therefore, data collected over long periods of time are required. It is important to observe retrospective data, if possible, and to define a baseline, against which to measure change that is attributable to global climate change [7].

Climate change environmental health indicators need to be both scientifically valid and politically relevant, therefore they should be [7,25,26]:

- Credible—based on a known link between climate and health
- Specific—directly related to a specific issue of climate change and health concern
- Actionable—related to climate/environmental/health conditions that are amenable to adaptive actions
- Sensitive to changes in climate and less sensitive to alternative (non-climate) explanations
- Relevant to an issue of policy or practical concern
- Sustainable—able to provide data for the next 20–30 years
- Consistent and comparable over time and space
- Scalable—capable of being used at different scales
- Robust and unaffected by minor changes in methodology, scale or data
- Unbiased and representative of the conditions and area of concern
- Explicit—identify specific adaptation responses
- Accurate—based on data of a known and acceptable quality
- Understandable, applicable, and acceptable to stakeholders and potential users
- Measurable—based on available data and manageable methods with retrospective data available to provide a baseline, against which change can be measured
- Cost-effective—capable of being constructed and used at an acceptable cost-benefit ratio
- Selective—in that they help to prioritize key issues in need of action
- Available in a timely manner.

Whilst it is recognized that few indicators can fulfill all of these criteria, the first four are considered to be essential. The weighting given to specific criteria may vary between indicators depending on their intended purpose [25].

## 4. Frameworks

In developing and presenting EHIs it is important to do so in a systematic structured framework to enable consistent monitoring and interpretation. Given the complexity of environmental health issues, it is beneficial to use a framework to develop and structure EHIs [25]. A framework provides a systematic approach that aids interpretation of these complex environmental health issues by demonstrating links or relationships between the environment and human health. The main role of a framework is to organize the concepts, ideas, and notions of a subject meaningfully [27]. They can also help ensure the selection of a relevant and balanced range of EHIs and help to recognize and interpret complicated links between them [25].

Robust frameworks have:

- Conceptual clarity and scope—ensuring that the framework covers all key concepts and includes logical and plausible links
- Flexibility—to allow for consideration of the issue at any stage or component of the framework
- Balance—the framework accommodates issues with an environmental or health emphasis equally well
- Usability—the framework lends itself to a viable methodology for developing suitable indicators.

Developing EHIs requires identifying links between environmental factors and human health outcomes, therefore, a framework that groups indicators into determinants and outcomes is useful. Such an approach is much more informative than simply presenting indicators as a list [28].

It is important when developing and utilizing EHIs to adopt a framework using an ecosystem health approach. An ecosystem health approach allows analysis of the whole ecosystem, extending the understanding of the nature and sources of human health and disease [29,30]. This approach is about understanding the relationship between humans and their ecosystems and the recognition of disrupted ecosystems as an environmental exposure variable to which the whole population is ultimately exposed [29]. Within the broad context of ecosystem health it is important to recognize that for humans the ecosystem includes cultural, social, economic, and political variables. Therefore, an ecosystem health approach allows for more explicit links between the state of the environment and human well-being. Identification of these explicit linkage pathways (ecological linkage mechanisms) within the broader hazard surveillance category is necessary as they introduce another order of hazard surveillance that may not otherwise be obvious as they are indirect (e.g., biodiversity) [31,32].

Various frameworks have been developed in the areas of environment, health, environmental health, and indicators. Here we review these frameworks in respect to their attributes for developing EHIs to measure and monitor the impacts of climate change on human health (Table 1).

### 4.1. Pressure-State-Response Framework

The Pressure-State-Response (PSR) framework was developed in the early 1990s by the Organization for Economic Co-operation and Development [33] to promote a common set of environmental performance indicators.

Early work on environmental indicators often focused on the ‘state’ of the environment, that is monitoring physical changes in the natural environment. This approach provides information that something is going wrong, but it does not provide information on why something is going wrong and what is being done to ameliorate the situation. Therefore, this approach was extended to include an evaluation of human activities that resulted in change—the ‘pressure’ and social ‘responses’ to try to control the impact of damaging human activities [34].

**Figure 1:**
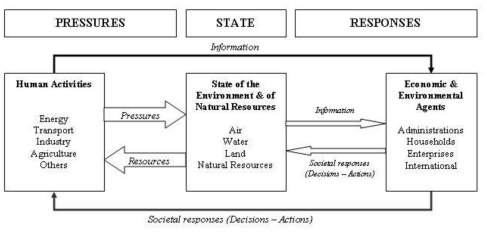
Pressure-State-Response framework (reproduced with permission from [33], published by OECD, 1993). Human activities exert pressures on the environment and change its quality and the quantity of natural resources (“State” box). Society responds to these changes through environmental, economic, and sectoral policies (“Societal responses”) intended to prevent, reduce, or mitigate pressures, and/or environmental damage. Social responses form a feedback loop to pressures through human actions.

Early causal frameworks for environmental statistics tended to make one-to-one linkages among particular stresses, environmental changes, and societal responses. The PSR framework as illustrated in Figure 1 is based on a concept of causality. The PSR framework is widely used but is continually evolving. Development of the PSR framework was based on an environmental economics model and was not intended to describe the links between cause and effect in detail [35]. One of the main problems has been differentiating between pressure and state indicators, and the need to expand the framework to deal more specifically with the need to describe sustainable development. A criticism of the PSR is that it tends to suggest linear relationships in the interaction between human activity and the environment [33]. A useful adaptation of the framework by the Commonwealth of Australia [36] was to incorporate circular feedback loops. Other adaptations have included broader driving forces and their impacts, resulting in the pressure-state- impact-response framework, which takes into account human health, ecosystem, and social-economic impacts [37]. Overall, the PSR framework has been considered unsuitable for describing human health linkages [38].

### 4.2. Driving Force-State-Response Framework

A development of the PSR framework was the Driving force-State-Response (DSR) framework adopted by the United Nations Commission on Sustainable Development. The following components make up the DSR framework for sustainable development: Driving force—human activities, processes, and patterns that impact on sustainable development; State—the ‘state’ of sustainable development; Response—policy options and other responses to changes in sustainable development. The term ‘pressure’ is replaced with ‘driving force’ to accommodate more accurately the addition of social, economic, and institutional indicators. The term ‘driving force’ also allows for the impact on sustainable development to be positive or negative as is often the case for social, economic, and institutional indicators.

State of the environment indicators can be used to bring scientific findings from the field and laboratory to the general public and decision-makers. To be effective, the indicators should be aimed at an explicit target group. A set of indicators should not only provide information on the development in specific environmental problem areas, but also give a general impression of the state of the environment. Ideally, a set of indicators is a tool to reduce a large quantity of data to a simpler form, while still retaining essential meaning for the question being asked [34].

### 4.3. Driving Force-Pressure-State-Impact-Response Framework

A further development of the PSR and DSR frameworks is the European Union-developed Driving force-Pressure-State-Impact-Response (DPSIR) framework, which provides an overall mechanism for analyzing environmental problems. Driving forces—such as industry and transport, produce; Pressures—on the environment, such as polluting emissions, which then degrade the; State—of the environment, which then; Impact—on human health and ecosystems, causing society to; Respond—with various policy measures, such as regulations, information and taxes (these can be directed at any part of the system).

All three of the above frameworks (PSR, DSR, and DPSIR) are primarily focused on the environment and were designed to develop environmental indicators (Table 1). The DPSIR framework is the only one of these that considers the effects of the environment on human health, however, they are not the primary focus. These frameworks do not describe the exposure route (links between cause and effect) in detail and only the DPSIR framework describes distal causal factors in detail by introducing both driving force and pressure elements to the framework. Due to the limited description of the exposure route, the PSR and DSR frameworks cannot identify multiple entry points for actions. A further criticism of these frameworks is their tendency to portray the interaction between human activity and the environment as a linear relationship. Due to these limitations the PSR, DSR, and DPSIR frameworks are considered unsuitable for describing the linkages between the environment and health in detail, and therefore they cannot provide the guidance necessary to develop EHIs to measure and monitor the impacts of climate change on human health.

### 4.4. Burden of Disease Framework

The Burden of Disease (BoD) framework is used to integrate, validate, analyze, and disseminate fragmentary information on the health of populations so that it is truly useful for health policy and planning [39]. The purpose of the framework is to provide a quantitative measure of health status by determining how much ill health can be attributed to a specific risk factor. Two fundamental concepts of the BoD framework are: (i) attributable burden of disease—quantification of disease levels caused by human exposure to a particular risk factor; and (ii) avoidable burden of disease—to estimate the effects that reductions in the population exposure to these risk factors would have [40].

With minor adjustment, the BoD framework may make the concepts of attributable and avoidable burden principally applicable to climate impact assessments, but practical applicability may be limited by insufficient knowledge about future climate change and the associated changes in regional health risks [40]. This framework is not designed to develop indicators and it does not describe the exposure route or distal causal factors in detail (Table 1). In addition, it does not explicitly identify or include entry points for actions. Therefore, the BoD framework does not provide the guidance necessary to describe environment and health linkages in detail, and to develop EHIs to measure and monitor the impacts of climate change on human health.

### 4.5. Millennium Ecosystem Assessment-Ecosystems Services

The Millennium Ecosystem Assessment (MEA) was initiated in 2001 to assess the consequences of ecosystem change for human well-being and the scientific basis for action needed to enhance the conservation and sustainable use of those systems and their contribution to human well-being [41].

**Figure 2:**
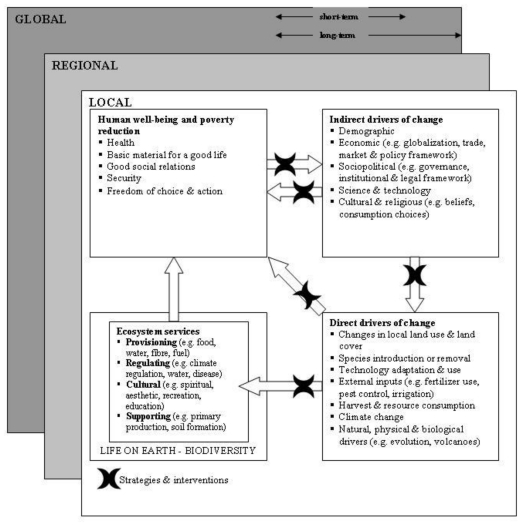
Millennium Ecosystem Assessment framework (reproduced with permission from [42], published by WHO, 2005). Changes in drivers that indirectly affect ecosystems (upper right box) can lead to changes in drivers that directly affect ecosystems (lower right box). The resulting changes in the ecosystem (lower left box) cause ecosystem services to change and thereby affect human well-being (upper left box). These interactions can take place at more than one scale, across scales, and across different timescales. Actions can be taken either to respond to negative changes or to enhance positive changes at almost all points in the framework (cross bars).

The MEA framework illustrated in Figure 2 lists the issues addressed in the MEA and shows their interrelationships. It cannot, of course, portray the complexity of these interactions in their temporal and spatial domains. This framework places human well-being as the central focus while recognizing that biodiversity and ecosystems also have intrinsic value and that people make decisions concerning ecosystems based on considerations of both well-being and intrinsic value [41].

As the MEA framework focuses on a specific system involved in the causal path ( *i.e.*, ecosystems) rather than a specific driver (*i.e.*, climate change) it cannot generally be applied to assessments of climate change impacts on health [41] (Table 1). Furthermore, the framework does not describe distal causal factors or the exposure route in detail and it was not designed to develop indicators. Therefore, the MEA framework like the BoD, is not suitable for developing EHIs.

### 4.6. Causal Webs

**Figure 3:**
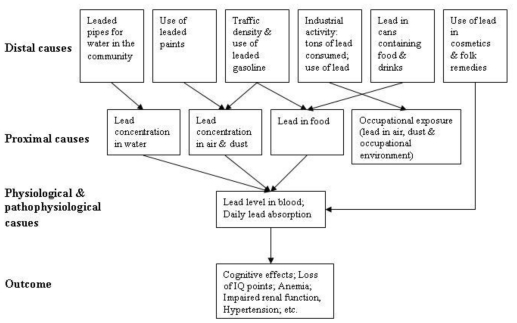
Causal web for chronic exposure to lead (reproduced with permission from [44], published by WHO, 2000). A causal web is a hierarchical cause-to-effect framework, categorizing three levels of risk factors: distal, proximal, and direct (physiological) causes. Distal causes operate through proximal, then direct causes on disease outcome.

Developed by MacMahon *et al.* [43], a causal web is a hierarchical cause-to-effect model that represents relationships among risk factors, and between risk factors and disease outcomes. As illustrated in Figure 3 a causal web shows a hierarchy of causes, categorizing three levels of risk factors: distal, proximal, and direct (physiological) causes. As the cause becomes more proximal to a disease outcome, a more direct analytical relationship is expected with the health outcome. Causal webs have been widely used in environmental health and have been applied to the effects of climate change on infectious diseases [44].

In principle, causal webs can be used to illustrate the etiology of any health problem, although they do not adequately differentiate between anthropogenic causal factors of a disease and natural baseline conditions as these are both categorized as ‘distal causes’. Differentiating between the two is important for modeling and the development of interventions. Therefore, the narrow categorization of causal factors into distal, proximal, and direct causes may not always be applicable when representing the complexity of the effects of climate change on health (Table 1). This approach would need to be extended to adequately describe the causal structure of different climate-sensitive health effects [40]. Although the framework could be modified to describe the causal structure of different climate-sensitive health effects, it was not designed to develop indicators and it does not explicitly identify or include entry points for actions. Therefore, the causal web framework does not provide the guidance necessary to develop EHIs to measure and monitor the impacts of climate change on human health.

### 4.7. Driving Force-Pressure-State-Exposure-Effect-Action Framework

**Figure 4:**
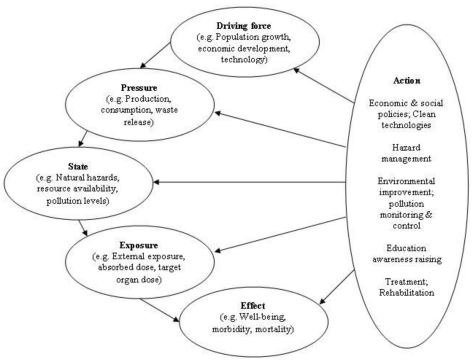
Driving force-Pressure-State-Exposure-Effect-Action framework (reproduced with permission from [14], published by WHO, 1999). The framework describes the environmental health chain through the following components: Driving force (anthropogenic)—factors that motivate and push the environmental process involved. Pressure (on the environment)—are normally expressed through human occupation or exploitation of the environment. State (of the environment)—status of the environment. Exposure (of humans *i.e.*, interaction between the environment and humans)—take place when humans are exposed to environmental conditions. Effect (in humans)—health effects from exposure to the environmental hazard. Action—policies or interventions aimed at reducing or avoiding health effects, they can be aimed at any point in the framework.

In the early 1990s the WHO developed a framework that incorporates transparent linkages between various one-dimensional environment or health indicators and places the focus on public health. As illustrated in Figure 4 the Driving force-Pressure-State-Exposure-Effect-Action (DPSEEA) framework for EHIs shows the link between exposures and health effects as determined by many different factors operating through a chain of events, and clearly shows the many entry points for interventions (Table 1). The framework describes the cause-to-effect chain through Driving forces, Pressures, State, Exposure, Effects and Actions, and provides a framework for analyzing interrelated factors that impact on human health [45].

The DPSEEA framework was designed to support decision making on actions to reduce the burden of disease by describing environmental health problems from their root causes through to their health effects, and by identifying areas for intervention [38,46]. It is a hierarchical approach that links measurable indicators to environmentally caused diseases, and displays the various levels of action that can be undertaken to reduce environmental health impacts. Analytical EHIs that quantify the impact at each step in the causal chain are particularly useful as they would highlight where an intervention aimed at protecting human health would be the most effective [13].

A primary strength of the DPSEEA framework is that it clearly identifies the many different intervention points along the environmental health causal chain [25]. Thus, the framework can be utilized to design and target interventions, as well as to monitor their performance. Furthermore, the DPSEEA framework works well for risks associated with environmental pollution where the chain from pollution source through to health effect is evident, providing a powerful tool that can demonstrate the links in the causal chain from hazard through to health effect [14]. The framework also incorporates the high level drivers and pressures leading to the state of the environment and exposures, often not addressed by other frameworks. Combining the ability to articulate the causal chain and the high level drivers makes the DPSEEA framework particularly useful in designing interventions further up the causal chain that may be more cost effective in improving health outcomes. The DPSEEA framework can also be applied to many perceived or psychological health effects brought about by fear of a hazard rather than its actual eventuality [14].

**Figure 5:**
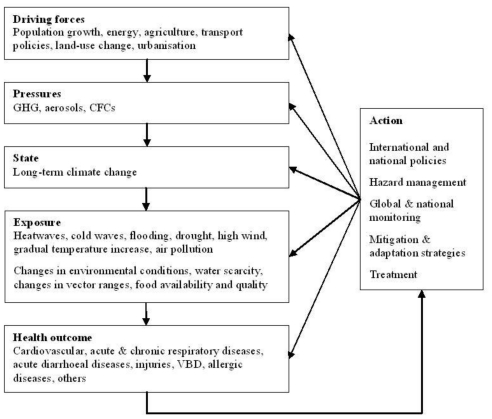
Driving force-Pressure-State-Exposure-Effect-Action framework applied to climate change [7].

Carneiro *et al.* [47] found that the framework proved useful for the analysis of complex environmental health issues as it addresses all of the complex levels from economic and social dynamics to environmental response, and human health. They also identify it as more efficient in analysis of these issues compared with other cause-to-effect frameworks [47]. A further advantage of the DPSEEA framework is its flexibility and applicability [48], it should be seen as an aid that can be adapted and modified according to requirements. There are several examples of how the framework has been successfully validated [47], adapted and applied to drinking water [49] and acute events (road accidents) [50], and to allow for contextual considerations [51]. The DPSEEA framework has also been adopted in a proposal for monitoring health impacts of climate change in Europe. Figure 5 illustrates how the DPSEEA framework can be adapted to address the potential health impacts of climate change [7].

Some authors have identified the framework as being less effective for representing physical risks, including natural hazards or technology [14,25]. They also highlight that the framework would have limitations if understood in a linear form, thereby not fully representing the complex associations between exposures and health outcomes, particularly, the importance of complex contextual considerations, such as, socio-environmental contexts and how they relate to underlying environment and health issues (e.g., the impacts of societal and behavioural influences on exposures and outcomes) [25,40,45,47,51]. This highlights the need to avoid simplifying environment and health relationships to linear links and to represent the actual complexity of these associations by illustrating the many interactions that are reality. However, the framework developers intended that the DPSEEA framework would take the shape of an inter- linking web rather than a straight chain for environmental health problems, thereby demonstrating that for a number of driving forces multiple health effects may occur and these effects may be associated with multiple exposures [35]. In capturing these complex interactions it is important to obtain multi-disciplinary and multi-stakeholder involvement.

Füssel and Klein [40] reviewed a variety of conceptual frameworks, including the DPSEEA framework, for their applicability for adaptation policy assessments of climate change and health. In this review, the DPSEEA framework was evaluated as being less well suited to represent the complex and diverse causal web that links climatic, environmental, and social factors to human health and it is argued that to be useful for the identification and monitoring of climate-health indicators and for the development of response strategies to climate change it would need to be extended in a flexible way to include intermediate ecological indicators and relevant non-climatic confounding factors [40]. Therefore, the DPSEEA framework can provide the guidance necessary to develop EHIs to measure and monitor the impacts of climate change on human health.

### 4.8. Multiple Exposures-Multiple Effects Framework

**Figure 6:**
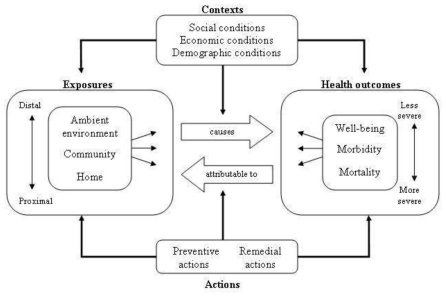
Multiple Exposures-Multiple Effects framework (reproduced with permission from [25], published by WHO, 2003). Exposures (left box) in different environmental settings lead to many different health outcomes (right box). Individual health outcomes may be linked back to many different exposures. Both exposures and health outcomes are affected by contextual conditions. Actions can be targeted at exposures or health outcomes or at underlying contextual factors.

The Multiple Exposures-Multiple Effects (MEME) framework is a further development of the DPSEEA framework for use in the context of children’s environmental health. This framework emphasizes the complex relationships between environmental exposures and child health outcomes. It is both a simplification and an extension of the DPSEEA framework. It is often difficult, in practice, to distinguish between the state and pressure elements of the DPSEEA framework. The MEME framework (Figure 6) combines the state, pressure, and exposure components under the general heading of exposure, recognizing that indicators of exposure may be assessed more or less directly, with state or pressure components often serving as proxies for the actual exposure [52]. The MEME framework also recognizes contextual factors (such as social conditions, economic development, and demographics) and their influence on both exposures and health outcomes [25]. The similarities of the MEME and DPSEEA frameworks mean that it is relatively simple to switch between them according to need.

The MEME framework emphasizes the complex relationships between the environment and health, including recognition of contextual factors (Table 1). However, it does not separate proximal (exposures) from distal (pressure and state) causes. The ability of a framework to distinguish proximal from distal causes is particularly useful for designing and applying interventions further up the causal chain. These higher level interventions may often be more cost effective in reducing negative health outcomes. Therefore, for complex environmental health issues (such as health effects due to climate change) it may be helpful to arrange the issues more formally as in the DPSEEA framework.

### 4.9. Environmental Public Health Indicator Framework

The Environmental Public Health Indicator (EPHI) framework was developed by the CDC and organizes indicators into a hazard, exposure, health effect, and intervention structure. The framework is based on concepts from Thacker *et al.* [53], which describe hazard, exposure, and outcome surveillance for environmental public health. It shows adapted structural components and concepts from the PSR framework for indicators of sustainable development and the DPSEEA framework for indicators of environmental protection and public health [54] (Table 1). Indicators are categorized as a hazard, exposure, health effect, or intervention:

- Hazard indicators: Conditions or activities that identify the potential for exposure to a contaminant or hazardous condition.
- Exposure indicators: Biologic markers in tissues or fluids that identify the presence of a substance or combination of substances that could harm an individual.
- Health effect indicators: Diseases or conditions that identify an adverse effect from exposure to a known or suspected environmental hazard.
- Intervention indicator: Programs or official policies that minimize or prevent an environmental hazard, exposure, or health effect.

Environmental public health indicators can be used to assess health status or risk as they relate to the environment. The best indicators are those that reliably predict the relationship between human health and the environment, are routinely collected, and have well-accepted definitions and data collection standards [54].

The EPHI framework like the DPSEEA and MEME frameworks, utilizes the concept of the environmental health chain. In the EPHI framework all environmental factors upstream from the point of exposure are included in the broad ‘hazard indicator’ category. As in the MEME framework, proximal causes (exposures) are not separated from distal causes (pressure and state) and thus the EPHI framework is not as useful for designing and applying interventions as the DPSEEA framework.

### 4.10. Health Impact Assessment

Health Impact Assessment (HIA) is used to judge the potential health effects of a policy, program or project on a population, particularly on vulnerable or disadvantaged groups. Recommendations are provided for decision-makers and stakeholders, with the aim of maximizing the proposal’s positive health effects and minimizing its negative health effects [55].

Most applications of HIA, to date, have tended to be relatively local and limited in scope [25]. However, there are examples of HIA methods being applied to climate change [56,57]. Patz *et al.* [57] argue that the HIA framework provides a comprehensive and policy-relevant approach to improve decisions on climate change and health policy (Table 1). The HIA framework can be used to help measure co-benefits alongside averted disease-specific risks for a more comprehensive decision support tool and that considers health equity. Nelson [56] illustrated the application of the disability-adjusted life year index in an HIA to assess the health impacts of global climate change in Bangladesh. They found that this tool could be used to compare and rank hazards and therefore this level of analysis may be sufficient to rank risks.

As demonstrated by Nelson [56] and Patz *et al.* [57], the HIA framework can be applied to assess the impacts of climate change on human health. However, it is not specifically a tool for developing indicators and does not explicitly identify or include entry points for actions. Therefore, the framework cannot provide the guidance necessary to develop EHIs to measure and monitor the impacts of climate change on human health.

### 4.11. Integrated Environmental Health Impact Assessment

**Figure 7:**
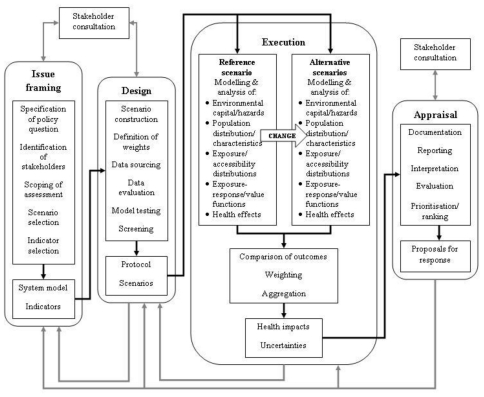
Integrated Environmental Health Impact Assessment framework [58]. This is a four-stage process, comprizing of: Issue framing—defines the problem/purpose for assessment. This focuses and limits the scope of assessment and management options. Design—purpose is to convert the conceptual model devised during issue framing into a detailed protocol for assessment. Execution—the core of the assessment process. Appraisal—synthesis and interpretation of results.

Integrated Environmental Health Impact Assessment (IEHIA) is illustrated in Figure 7 and is defined as a means of assessing health-related problems derived from the environment and health-related impacts of policies and other interventions that affect the environment, in ways that take account of the complexities, interdependencies, and uncertainties of the real world. The framework takes a broad, all encompassing view of both health and the environment and acknowledges that the whole environmental health system is subject to external influences that act as forces for change within the system.

Therefore, IEHIA involves analyzing the impacts of the environmental capital and hazards within the context of these changing external influences [58]. The IEHIA framework incorporates and links several assessment methods including traditional risk assessment, comparative risk assessment, and HIA. It combines a qualitative approach for issue framing and selection and design of appropriate methods of assessment, and a quantitative approach for carrying out integrated assessments of complex issues. Both of these approaches have limitations. The qualitative approach involves a strong participatory approach and therefore issues of stakeholder involvement are inherent. The quantitative approach involves modeling and analyses of complex, multivariate systems, often with limited data and knowledge. Primary challenges of such an integrated approach are how to cope with the non-linearity and multi-causality of environmental health issues [58].

Like the HIA framework, an IEHIA approach could be applied to assess the impacts of climate change on human health (Table 1). However, it is not specifically a tool for developing EHIs and does not explicitly identify or include entry points for actions. Therefore, IEHIA framework would provide limited guidance for developing EHIs to measure and monitor the impacts of climate change on human health.

## 5. Conclusions

The development and utilization of EHIs to measure and monitor the impacts of climate change on human health is a challenging environmental health issue. Therefore, it is important to adopt a framework that provides a systematic approach to aid interpretation of the complex interactions by demonstrating links or relationships between the environment and human health. Such a framework with an ecosystem health approach recognizes that disrupted ecosystems are an environmental exposure variable to which the whole population is ultimately exposed. This approach enables the identification of explicit links (both direct and indirect) between the state of the environment and human well-being.

It is difficult for any framework to fully represent the complex interactions involved in assessing the impacts of climate change on human health. These interactions can occur both in varying time and space and are often non- linear in nature. That said, frameworks should be seen as tools that can be modified according to requirements. Also the purpose for which the framework is to be utilized should not be forgotten. In our review we are interested in frameworks that can be used for developing EHIs to assess and monitor the impacts of climate change on health and aid in the development of interventions.

We consider the DPSEEA framework to be the best suited for developing EHIs to assess and monitor human health vulnerability, to aid in the design and targeting of interventions, and measure the effectiveness of climate change adaptation and mitigation activities. Taking this further, DPSEEA EHIs that incorporate the monitoring and integration of human, animal, and environmental (including plants) health data have the potential to triangulate and identify emerging ecological problems. In essence this holistic approach could act as a warning system for ecosystem disruption and be used to identify interventions for the preservation of ecologic, human, and animal health. If the linkage mechanisms are explicit and adequately represented then interventions can be applied higher up the causal chain than would have been possible based on environmental monitoring or health surveillance alone (e.g., Cook *et al.* [59]). Implementation of such interventions would not only improve ecological well-being, but in turn would reduce the resultant burden of disease.

## Figures and Tables

**Table 1:** Attributes of the frameworks.

Framework attributes^a^	Framework										
	PSR	DSR	DPSIR	BoD	MEA	Causal web	DPS EEA	MEME	EPHI	HIA	IEHIA
**Designed for indicators**	Yes	Yes	Yes	No	No	No	Yes	Yes	Yes	No	Yes
**Includes environment & health components**	No	No	Yes	Yes	Yes	Yes	Yes	Yes	Yes	Yes	Yes
**Utilizes causal chain approach**	Yes	Yes	Yes	No	Yes	Yes	Yes	Yes	Yes	Yes	Yes
**Describes distal causal factors in detail**	No	No	Yes	No	No	No	Yes	No	No	Yes	Yes
**Explicitly includes exposure route**	No	No	No	No	No	Yes	Yes	Yes	Yes	Yes	Yes
**Explicitly includes actions/interventions**	Yes	Yes	Yes	No	Yes	No	Yes	Yes	Yes	Yes	Yes
**Explicitly includes multiple entry points for actions/interventions**	No	No	Yes	No	Yes	No	Yes	Yes	Yes	No	No
**Can be adapted to measure & monitor the impacts of climate change on human health**	No	No	No	Yes	No	Yes	Yes	Yes	Yes	Yes	Yes

*Abbreviations*: PSR Pressure-State-Response; DSR Driving force-State-Response; DPSIR Driving force-Pressure-State-Impact-Response; BoD Burden of Disease; MEA Millennium Ecosystem Assessment; DPSEEA Driving force-Pressure-State-Exposure-Effect-Action; MEME Multiple Exposure-Multiple Effect; EPHI Environmental Public Health Indicators; HIA Health Impact Assessment; IEHIA Integrated Environmental Health Impact Assessment.

a Refers to historically used and potential attributes of a framework.
